# Reduced Spontaneous Eye Blink Rates in Recreational Cocaine Users: Evidence for Dopaminergic Hypoactivity

**DOI:** 10.1371/journal.pone.0003461

**Published:** 2008-10-22

**Authors:** Lorenza S. Colzato, Wery P. M. van den Wildenberg, Bernhard Hommel

**Affiliations:** 1 Institute for Psychological Research & Leiden Institute for Brain and Cognition, Leiden University, Leiden, The Netherlands; 2 Amsterdam Center for the Study of Adaptive Control in Brain and Behaviour (ACACia) Psychology Department, University of Amsterdam, Amsterdam, The Netherlands; University of Granada, Spain

## Abstract

Chronic use of cocaine is associated with a reduced density of dopaminergic D2 receptors in the striatum, with negative consequences for cognitive control processes. Increasing evidence suggests that cognitive control is also affected in recreational cocaine consumers. This study aimed at linking these observations to dopaminergic malfunction by studying the spontaneous eyeblink rate (EBR), a marker of striatal dopaminergic functioning, in adult recreational users and a cocaine-free sample that was matched on age, race, gender, and personality traits. Correlation analyses show that EBR is significantly reduced in recreational users compared to cocaine-free controls, suggesting that cocaine use induces hypoactivity in the subcortical dopamine system.

## Introduction

In the long term, daily or chronic use of cocaine is associated with a reduction in dopamine D2 (DAD2) receptors in the striatum [1] and with hypoactivity dysfunctions in the lateral prefrontal cortex, the anterior cingulate cortex, and the orbitofrontal cortex [2], [3], [4]-areas that play a key role in the regulation of human cognition and the control of goal-directed behaviour [5], [6]. Findings that cocaine-dependent individuals show impaired abilities to inhibit overt responses [7] support the notion that cocaine, through its selective effect on DAD2 receptors [1], modulates cognitive control in general [8] and response inhibition in particular [9].

Recent studies suggest that recreational users of cocaine, who do not meet the criteria for abuse or dependence but take cocaine on a monthly basis (1 to 4 gram, commonly consumed in only a few sessions, so that the peak use [bingeing] often equals this monthly dose) exhibit cognitive symptoms similar to those of chronic users. Colzato, van den Wildenberg, and Hommel [10] showed that recreational use impairs response inhibition to a degree that is proportional to lifetime cocaine exposure. This indicates that the inhibitory deficit increases as a function of the history of cocaine use [2], [11]. Colzato and Hommel found that, relative to a sample of cocaine-free controls, recreational users show increased costs in switching between different tasks [12] and no reliable inhibition of return (IOR) [13]-the (commonly robust) phenomenon of slowed responding when attention needs to return to a previously attended location [14].

The observation that recreational use of cocaine is associated with impairments in response inhibition, cognitive flexibility, and IOR—all functions that are assumed to rely on dopaminergic pathways [9], [15], [16]—suggests that recreational use is sufficient to hamper the dopaminergic support of cognitive control functions. However, the relation between mental control functions and cocaine is not straightforward because pre-existent neuro-developmental factors cannot be excluded. Recent studies showed, indeed, that those individuals with pre-existing lowered D2 receptor densities are more at risk to use cocaine and to become addicted [17] and that chronic users may suffer from pre-existing inhibitory deficits [18].

The present study sought for converging evidence supporting the hypothesis that recreational use of cocaine is associated with impairments in the dopaminergic system. The dependent measure of interest for our purposes was the spontaneous eye-blink rate (EBR), a well established clinical indicator [19] that is generally interpreted as a marker of dopaminergic functioning in the striatum [21]–[24, but see 20]. First, the functional link between the striatum and EBR is supported by clinical observations in patients with DA-related dysfunctions: Whereas EBR is elevated in schizophrenic patients with increased dopaminergic activity in the striatum [25], EBR is reduced in Parkinson's patients who suffer from a loss of nigrostriatal dopaminergic cells [26]. Second, the link is sustained by pharmacological studies in nonhuman primates showing that direct DA agonists and antagonists respectively increase and decrease blink rates [23]. Third, a genetic study in humans demonstrated a strong association between EBR and the DRD4/7 genotype, which is related to the control of striatal DA release [27].

Following this reasoning, and given that cocaine use is associated with reduced D2 receptors [1], we expected that recreational users would show a significantly *reduced* EBR compared to a matched sample of cocaine-free controls. Second, given that the magnitude of cognitive impairments in response inhibition seems be proportional to the history of cocaine use [10], we expected a negative correlation between cocaine exposure and EBR, indicating that the magnitude of dopaminergic malfunction is proportional to life-time cocaine use.

## Results

Data were examined using Brain Vision Analyzer (Brain Products™ GmbH, Munich, Germany; http://www.brainproducts.com/products/analyzer/index_analyzer.html). We defined an eyeblink as a voltage change of 100 µV in a time interval of 500 ms. EBR per minute was significantly lower, *t*(22) = −3,42, *p*<.05, in recreational users, (M = 9.3; SD = 5.9), than in cocaine-free controls (M = 17.1; SD = 5.3)—a possible indication of hypoactive subcortical dopamine systems in cocaine users.

Second, we tested whether alcohol and nicotine consumption and IQ contributed to the effect on eyeblink measures. However, an ANOVA with group (recreational cocaine users vs. cocaine-free controls) as independent variable and monthly drinks, monthly cigarettes and IQ as covariates did not suggest such contribution: the effects of the covariates were far from significant, *F*<1, while the group effect remained reliable, *F*(1, 20) = 4.38, *p*<.05.

Third, to test whether the magnitude of dopaminergic malfunction is proportional to the amount of cocaine consumed, we computed Spearman's Rho correlation coefficients between individual lifetime cocaine exposure, highest regular frequency, peak and monthly cocaine dose, and EBR per minute. Peak correlated negatively with EBR, *r*(12) = −.67, *p*<.05, while lifetime cocaine exposure, *r*(12) = −.12, highest regular frequency, *r*(12) = −.50, and monthly cocaine dose, *r*(12) = −.28, did not. Hence, the larger the amount of cocaine consume in a 12-h period, the lower the eye blink rate and, by inference, the more pronounced the dopaminergic malfunction.

## Discussion

Our finding shows that the spontaneous EBR, a functional marker of dopaminergic functioning [19], is significantly reduced in recreational cocaine users as compared to cocaine-free controls. Moreover, the degree of this reduction corresponds to the peak of cocaine consume, suggesting that higher peaks are associated with increased dopaminergic hypoactivity. The pattern observed fits with the notion that chronic use of cocaine produces a reduced functioning of DAD2 receptors in the striatum [1]. Apparently, then, even small doses of cocaine are associated with a reliable change in striatal dopaminergic functioning.

As Bolla et al. [2] previously pointed out, the effects of chemicals on the central nervous system are generally subtle and may occur only at higher doses. This is why Bolla and colleagues consider it critical to compare individuals with highest exposure to individuals with low or no exposure. According to these authors, exposure duration is critical to the development of neurocognitive effects after organic and inorganic lead exposure, whereas exposure intensity is more critical for the development of neurocognitive effects following exposure to organic solvents. Along these lines, Cascella et al. [28] found that the amount of alcohol consumption per session at the time of peak use (bingeing) was a better predictor of brain atrophy than current alcohol consumption. According to this logic, it makes sense that we found cocaine bingeing to be a better predictor of EBR and the hypothesized underlying dopaminergic malfunction than current cocaine consumption.

In contrast to numerous previous studies on chronic cocaine use (see [29]), the design of our study allows us to reject a number of alternative accounts of our observations. Participants were screened for several psychiatric disorders and matched for age, race, IQ, gender, alcohol consumption, and personality traits, which rules out interpretations in terms of pre-existing psychiatric disorders (such as schizophrenia, ADHD, and obsessive compulsive disorder) that are known to affect the dopaminergic system [30], [31], [32] This finding is in line with evidence that Parkinson's patients, who suffer from loss of nigrostriatal dopaminergic cells, show cognitive impairments and lower EBR than controls [26]. Particularly important was the matching of the personality traits: From previous studies on EBR it is known that higher scores on psychoticism scales are associated with elevated EBR [33]. Even though our recreational users also used other recreational drugs in the past, marijuana and MDMA in particular, we doubt that our results can be attributed to the use of these substances: MDMA is associated primarily with impairments in serotonine supply [34] and the long-term after-effect of cannabis (in contrast to effects in current users) is not known to be neurotoxic [35].

To conclude, the findings obtained in this study are important in suggesting a selective dopaminergic malfunction resulting from, or at least connected with, the consumption of rather small doses of cocaine. However, as mentioned earlier, recent evidence suggests that subjects with pre-existing lowered D2 receptor densities run a higher risk to use cocaine and to become addicted [17]. The status of cocaine use as cause versus effect in the context of this and other disorders of impulse control, such as ADHD and pathological gambling, is still uncertain and more research is needed to determine the relative contributions of cocaine consumption and other pre-existing constellations to impairments of the dopaminergic system.

## Materials and Methods

### Participants

Twenty-four young healthy adults served as participants, consisting of two groups of 12 recreational users of cocaine and 12 cocaine-free controls. One recreational cocaine user received course credit while the other participants received a financial reward. The sample was drawn from adults in the Leiden, Rotterdam, and Delft metropolitan areas, who volunteered to participate in studies of behavioural pharmacology. Participants were recruited via notices posted on community bulletin boards and by word of mouth.

Following Colzato et al. [12], we made sure that the users met the following criteria: 1) a monthly consumption of cocaine (1 to 4 gram) by snorting route for a minimum of two years; 2) no Axis 1 psychiatric disorder (DSM-IV [36]), including ‘substance abuse’; 3) no clinically relevant medical disease; 4) no use of medication; 5) no family history of alcoholism and/or substance use disorder. Cocaine-free controls met the same criteria except that they reported no history of past or current cocaine use. Subjects were selected by means of a phone interview by a research assistant with the M.I.N.I. [37], a brief diagnostic tool that screens for several psychiatric disorders including, among others, schizophrenia, depression, mania, and obsessive-compulsive disorder. Participants with a known history of psychopathology and those who were taking medication were excluded. The sample was obtained from a pool of approximately 35 potential volunteers who responded to the advertisement “recreational cocaine users and cocaine free controls” for studies conducted in our lab over the period of 6 months. Within this pool of potential volunteers, the most common reason for excluding an individual from the study was meeting psychiatric disorder (ADHD, Mania) or medication use.

In line with Colzato, van Wouwe, and Hommel [38], participants were asked to refrain from taking drugs for two days and from all caffeine containing foods and beverages for 12 hours prior to the experiment, not to consume alcohol on the night before the experimental session and to have a normal night rest. Subjects' compliance with the instruction was encouraged by taking a saliva sample (not further analyzed) at the beginning of the session (cf., [39]).

During the month that preceded testing, three out of twelve recreational users also smoked marijuana, while two reported to have taken one MDMA (ecstasy) tablet. The cocaine-free controls reported not having used any drug. Participants in the two groups were matched for race (100% Caucasian), age, gender, IQ (measured by Raven's Standard Progressive Matrices (SPM [40]), alcohol consumption, and personality traits psychoticism, extraversion, neuroticism, and social approval (measured by the Eysenck Personality Questionnaire Revised Short Scale [41]).

Demographic and drug use statistics are provided in Tables 1 and 2. In Table 2, t-tests were calculated to compare the demographic characteristic of the two groups. Written informed consent was obtained from all participants after the nature of the study was explained to them. Protocol and subject honoraries were approved by the institutional review board (Leiden University, Institute for Psychological Research).

**Table 1:** Demographic characteristics, EPQ-R scores, and use of other recreational drugs.

	Controls	Recreational users	Significance level
N (M∶F)	12 (10∶2)	12 (10∶2)	ns
Age (years)	28.3 (5.2)	27.5 (5.6)	ns
Raven IQ	124.2 (4.5)	116.3 (3.7)	ns
Monthly drinks	60.0 (61.6)	90.5 (61.6)	ns
Monthly cigarettes	144.1 (291.8)	240.8 (196.7)	**
Lifetime exposure cannabis	-	483.3 (174.9)	**
Lifetime exposure MDMA	-	65.2 (51.8)	**
Psychoticism trait	3.5 (1.0)	4.4 (1.6)	ns
Extraversion trait	9.9 (2.2)	10.7 (1.2)	ns
Neuroticism trait	3.1 (2.9)	5.1 (2.7)	ns
Social Approval trait	4.5 (2.1)	3.2 (4.5)	ns

Standard deviation in parentheses. Ns, Non-significant difference; Raven IQ, IQ measured by means of the Raven Progressive Matrices; Monthly drinks, monthly number of standard alcoholic drinks; Monthly cigarettes, monthly standard cigarettes smoked; Lifetime exposure cannabis, lifetime consumption of joints; Lifetime exposure MDMA, lifetime consumption of MDMA (ecstasy) tablets; Personality traits measured by means of the Eysenck Personality Questionnaire Revised Short Scale.

** *p*<0.01.

**Table 2:** Self-reported use of cocaine. Standard deviation in parentheses.

Sample	Mean (SD)
Highest regular frequency (times per month)	3.5 (2.9)
Highest amount in a 12-h period (peak; grams)	1.30 (0.82)
Monthly grams	2.15 (1.18)
Lifetime exposure grams	270 (193)
Monthly money cocaine (Euro)	107.5 (58.9)

### Procedure and design

A BioSemi ActiveTwo system (BioSemi Inc., Amsterdam, The Netherlands) was used to record the EBR. Eyeblinks were recorded with two horizontal electrodes in the outer canthi of both eyes (HEOG) and two vertical electrodes in the infraorbital and supraorbital regions of the left eye (VEOG). EBR was recorded for 6-min eyes-open segments under resting conditions. The horizontal electro-oculogram, which records the voltage difference between electrodes placed lateral to the external canthi, was used to measure horizontal eye movements. The vertical electro-oculogram, which records the voltage difference between two electrodes placed above and below the left eye, was used to detect eye blinks. A low-pass filter of 40 Hz was applied and electro-oculogram signals, digitized at 512 Hz, were averaged offline. Given that spontaneous EBR is supposed to be stable during daytime but increases in the evening (8:30 p.m., as reported by [42]), all data were collected before 5 p.m. To minimize influences of demographic variables on EBR, we paid attention to match our subjects for age and gender, given that women exhibited a higher tonic EBR than men. Subjects were comfortably sitting in front of a blank poster with a cross in the centre, located about 1 meter from the subject. The subject was alone in the room and was instructed to look at the cross in a quiet relaxed state for six minutes.
